# Are there inequalities in the attendance at and effectiveness of behavioural weight management interventions for adults in the UK? An individual participant data meta-analysis

**DOI:** 10.1136/bmjph-2024-001382

**Published:** 2025-08-07

**Authors:** Jack M Birch, Julia Mueller, Sarah Damery, Stephen J Sharp, Rebecca A Jones, Michael P Kelly, Annie S Anderson, Nerys Astbury, Paul Aveyard, Rebecca J Beeken, Angela Craigie, Colin Greaves, Susan Jebb, Alex McConnachie, Kiran Nanchahal, Beth Stuart, Martin White, Simon J Griffin, Amy L Ahern

**Affiliations:** 1 MRC Epidemiology Unit, University of Cambridge, Cambridge, UK; 2 Institute of Applied Health Research, University of Birmingham, Birmingham, UK; 3 Department of Public Health and Primary Care, University of Cambridge, Cambridge, UK; 4 School of Medicine, University of Dundee, Dundee, UK; 5 Department of Primary Care Health Sciences, University of Oxford Nuffield, Oxford, UK; 6 School of Medicine, University of Leeds, Leeds, UK; 7 School of Sport, Exercise and Rehabilitation Sciences, University of Birmingham, Birmingham, UK; 8 Robertson Centre for Biostatistics, University of Glasgow, Glasgow, UK; 9 Department of Public Health, Environments and Society, London School of Hygiene & Tropical Medicine, London, UK; 10 Wolfson Institute of Population Health, Queen Mary University of London, London, UK

**Keywords:** Public Health, Preventive Medicine, Body Mass Index

## Abstract

**Objectives:**

Interventions for obesity that require individual behaviour change, such as behavioural weight management interventions, may contribute to health inequalities. We explored if there was evidence of inequalities in the attendance at and effectiveness of behavioural weight management interventions in adults.

**Design:**

Two-stage individual participant data meta-analysis.

**Data sources:**

Eligible studies were extracted from a previous systematic review and an updated search was completed on PubMed.

**Eligibility criteria:**

UK-based randomised controlled trials of behavioural weight management interventions suitable for use in primary care, published until 31 December 2021.

**Data extraction and synthesis:**

Multivariable regression analyses were conducted with weight at 12-month follow-up as the primary outcome and included an interaction between inequality characteristic and trial arm (control or intervention). Each model was adjusted for baseline weight, age and gender. Estimated interactions were combined across trials using a random-effects meta-analysis. Intervention attendance was defined as number of in-person sessions attended. Risk of bias was assessed using Cochrane’s RoB 2 tool.

**Results:**

Data from 13/16 eligible weight loss trials were analysed (complete case data n=5531 participants). The effect of the intervention on weight at 12 months was greater in male participants (−2.58 kg (95% CI −3.52 to 1.64)) than female participants (−1.71 kg (95% CI −2.79 to –0.63); p value for interaction=0.02, tau^2^=0) and greater for participants of white ethnicity (−2.74 kg (95% CI −4.30 to –1.19)), than those from an ethnic minority background (0.03 kg (95% CI −1.29 to 1.35); p interaction=0.04, tau^2^=0). Age, education, occupation, place of residence and household income did not significantly moderate effectiveness. We did not find evidence of inequalities in intervention attendance by ethnicity, occupation, gender/sex, area-level socioeconomic deprivation or age.

**Conclusions:**

Behavioural weight management interventions had smaller effects in people from ethnic minority backgrounds and larger effects in men. There was no evidence of other differences in intervention effectiveness or adherence. This is the first synthesis study to access individual participant data and quantitatively assess inequalities in these interventions. Future research should further explore reasons for differences in outcomes and consider how to prevent behavioural weight management interventions from potentially exacerbating health inequalities.

**WHAT IS ALREADY KNOWN ON THIS TOPIC**

- It has been suggested that interventions focusing on individual behaviour change, such as those commonly accessed by people living with overweight or obesity to manage their weight, may be susceptible to exacerbating health inequalities. Narrative evidence from previous systematic reviews has found some evidence to suggest there may be inequalities in the attendance at and effectiveness of behavioural weight management interventions in adults, but the evidence is not conclusive and has not been healthcare system specific.

**WHAT THIS STUDY ADDS**

- In this first individual participant data meta-analysis of inequalities in behavioural weight management interventions, we have shown that behavioural weight management interventions are less effective for people from an ethnic minority background.

**HOW THIS STUDY MIGHT AFFECT RESEARCH, PRACTICE OR POLICY**

- Behavioural weight management interventions that are currently commissioned may not be effective for those from minority ethnic groups. Future research should investigate the mechanisms behind the observed inequalities by ethnicity so that people with obesity from minority ethnic groups can be better supported to manage their weight in ways that are culturally appropriate.

## Introduction

 The prevalence of overweight and obesity, and of related comorbidities, is higher in those experiencing socioeconomic deprivation, having fewer years of education and in ethnic minority groups.^1^,^4^ The first line intervention for people living with overweight or obesity to help manage their weight is behavioural weight management interventions. These interventions are effective at promoting weight loss in people living with overweight or obesity and can reduce cardiometabolic risk factors such as total cholesterol, systolic blood pressure and haemoglobin A1c (HbA1c).^5^,^7^ It has been suggested that such interventions, which focus on individual behaviour change and require high individual agency to take effect, may be more effective in some population groups than others, thereby exacerbating health inequalities.^8 9^ These inequalities may occur across many individual characteristics which are captured in the PROGRESS-Plus framework (place of residence, race/ethnicity, occupation, gender/sex, education, socioeconomic status, social capital, plus other factors for which discrimination could occur such as age and sexual orientation).^10^

In a previous systematic review, we synthesised evidence on whether characteristics related to health inequalities moderated the uptake, attendance and effectiveness of behavioural weight management interventions in randomised controlled trials (RCTs).^11^ We found that most trials did not consider whether inequalities were present at any stage of the study. For those trials that did, it was not possible to meta-analyse the results, as the analyses performed in each trial were not uniform and there were significant differences in how the PROGRESS-Plus characteristics were captured between trials, especially when comparing trials from different countries. Consequently, the results were synthesised narratively. We found some evidence that intervention uptake, follow-up rates and attendance were higher in those considered more advantaged. However, this relied on typically underpowered inequality analyses conducted in individual trials. To address this limitation, we conducted an individual participant data meta-analysis (IPD-MA) using data from UK-based trials of behavioural weight management interventions. This approach allows variables that have been measured differently across trials to be harmonised and data uniformly analysed.^12^ Focusing on a single country reduces some of the heterogeneity in how the PROGRESS-Plus characteristics are captured, facilitating data harmonisation. These factors allow a more precise consideration of whether inequalities occur in the attendance at and effectiveness of these interventions.

In this IPD-MA, we sought to compare how the following outcomes differed by individual characteristics that can stratify health opportunities and outcomes (defined using the PROGRESS-Plus Framework): (1) the effectiveness of behavioural weight management interventions (defined as the difference in weight change between intervention and control), (2) the weight outcomes of those who have participated in a behavioural weight management trial (defined as weight change in the overall cohort) and (3) attendance (defined as the number of in-person sessions attended) at behavioural weight management interventions.

## Methods

### Search strategy and selection criteria

This IPD-MA was reported in accordance with the Preferred Reporting Items for a Systematic Review and Meta-analysis of Individual Participant Data (PRISMA-IPD) guidelines.^13^ Full details of the methods are published in the protocol.^14^

We included UK-based RCTs that evaluated behavioural weight management programmes that could feasibly be delivered in primary care. Relevant trials were extracted from a previous systematic review,^11^ and we performed an updated search on Medline to include trials published between 5 March 2020 (date of previous search, PROSPERO registration CRD42020173242) and 31 December 2021. This was completed by two authors independently. The search strategy (online supplemental table 1) from the US Preventive Services Taskforce systematic review of behavioural and pharmacotherapy interventions for obesity was adapted with the support of a University of Cambridge School of Clinical Medicine librarian to focus solely on behavioural interventions. The study inclusion criteria were:

1. Participants: Adults aged 18 years and over with overweight or obesity (body mass index (BMI)≥25 kg/m^2^ with no upper limit) who were deemed suitable (either by the applicable study team or healthcare practitioner) for weight loss or weight loss maintenance intervention. Participants may have additional risk factors such as hypertension, dyslipidaemia, impaired glucose tolerance or impaired fasting glucose. Studies were excluded if the population was not selected based on a weight-related measure, included participants with BMI <25 kg/m^2^, if participants were pregnant or selected based on having a chronic disease where weight loss is part of disease management, or if the intervention was targeted at parents to change the behaviour of children. Only studies conducted in the UK were eligible.
2. Interventions: Behavioural weight management interventions with the primary aim of supporting weight loss or weight loss maintenance. Studies were included if they were conducted in, or were applicable to, primary care. Interventions may have been delivered alone or as part of a wider multicomponent intervention targeting diet and nutrition, physical activity, sedentary behaviour or a combination of these. Studies of pharmacological and surgical interventions were excluded unless the trial included behavioural only intervention and control arms. Interventions were considered applicable to primary care if they were conducted in this healthcare setting or were widely available in the community at a national or regional level (such as commercial weight loss programmes, text-message and other digital-based interventions); examples of settings not relevant to primary care include interventions delivered in inpatient settings, or in residential care homes.
3. Comparators: Wait-list control, usual care or minimal intervention (such as generic print or electronic materials).
4. Outcomes: Studies must have collected data on weight at baseline and a time point between the 12-month or 18-month follow-up from baseline. We used the 12–18 months postintervention time point as this is the most frequently reported time point in the literature, including in the prominent systematic reviews in the area (including the US Preventive Services Task Force review our search strategy was based on).
5. Study designs: Randomised or cluster-RCTs.

We contacted the primary authors of the eligible trials by email (using contact details acquired from the primary trial publication) to invite them to contribute data from the trial and collaborate on this study. They were contacted twice, 2 weeks apart, and if there was still no reply, we contacted other listed authors to invite them to contribute data. The additional authors were also contacted twice, following which we made no further attempts to acquire the data. Once data sharing agreements were in place, data were shared via a secure file transfer protocol site. We received data in any format convenient to the trial authors and converted it into Microsoft Excel format where appropriate. Where it was not possible for individual-level data to be shared, we provided the statistical code so that collaborators could perform the first-stage analyses in their trial and email the results for inclusion in the meta-analysis.

### Risk of bias assessment

Cochrane’s RoB 2 tool for RCTs was used to assess the risk of bias of all included studies.^15^ Bias across six domains (the randomisation process, allocation concealment, participant and trial personnel blinding, blinding of outcome assessment, incomplete outcome data and selective reporting) was considered, with each domain being assigned a rating of ‘low risk’, ‘unclear’ or ‘high risk’. The overall risk of bias was determined by the lowest rating assigned to any of the six domains (eg, if one domain scored unclear and the remaining domains low risk, then the overall risk of bias would be assigned as unclear). This was completed by two researchers (JMB and RAJ); disagreements in risk of bias assessment were resolved by discussion.

### Data analysis

We requested individual participant data pertaining to outcomes (baseline weight, weight at 12 months and attendance) and the PROGRESS-Plus characteristics (place of residence, ethnicity, occupation, gender/sex, religion, education, social capital, socioeconomic status, plus other factors where discrimination may occur such as age and sexual orientation). Data were checked for quality by two researchers (JMB and JM), who determined whether sample size and descriptive summaries of PROGRESS-Plus related variables, baseline weight and weight change matched those reported in the primary trial publication. Where discrepancies occurred, trial authors were contacted for clarification. We describe the overall IPD-MA sample descriptively and present the overall difference in follow-up weight between the intervention and control groups. Differential attrition across the trials was assessed using ORs.

To answer our three research questions, all initial analyses used multivariable linear regression of complete case data to determine differences by individual characteristics that stratify health opportunities and outcomes (defined using the PROGRESS-Plus Framework) and controlled for gender/sex and age at baseline. The two analyses with weight as outcomes also controlled for weight at baseline.

The primary research question analysed differences in the effectiveness of behavioural weight management interventions (defined as the difference in weight change between intervention and control). For each trial, weight at 12-month follow-up was used as the outcome and PROGRESS-Plus characteristic and its interaction with the randomised group (intervention or control) as the exposures. Following the regression analyses, the estimated interaction parameters were then combined across trials using random effects meta-analysis. Heterogeneity was assessed using tau^2^ (summarising between-studies variance) and a 95% prediction interval (PI) indicating the range in which 95% of the true effects lie. Inconsistency was assessed using I^2^, indicating the proportion of total variability in the observed effects that was due to heterogeneity. Post hoc analyses, where differences in weight within each randomised group were calculated for each subgroup (eg, in men and women). When considering the issue of multiplicity, we decided against using a statistical adjustment—such as a Bonferroni correction—to adjust for multiple comparisons as such adjustments may inadvertently increase the risk of type II errors.^16^ The issue of multiplicity can be addressed by having a clear rationale for analyses based in evidence or theory for each performed test,^16 17^ which in this study is presented through the PROGRESS-Plus criteria. Each PROGRESS-Plus characteristic has a broad evidence base supporting its association with health inequalities.

For the analysis of differences in weight outcomes of those who had participated in a behavioural weight management trial (defined as weight change in the overall cohort) the association between the exposure (PROGRESS-Plus characteristic) and weight at 12-month follow-up additionally adjusted for assigned intervention (intervention or control). The estimated exposure/weight associations were combined across trials using random effects meta-analysis models with the restricted maximum likelihood (REML) estimation method. The SE from the pooled interaction estimate was used to derive the relevant CI.

Finally, for the analysis of differences in attendance of behavioural weight management interventions, we defined attendance as the percentage of offered sessions which were attended (in trials where there was >1 in person session). The association between each PROGRESS-Plus characteristic and attendance was estimated in a multivariable regression model. Each model was adjusted for age and sex, except where these were the exposure variable of interest. Associations were estimated for each subgroup in a trial. Estimated exposure/attendance associations were combined across trials using random effects meta-analysis (REML estimation method).

We conducted sensitivity analyses excluding studies for which we did not have access to the individual-level data but were sent the outcome results. We had also planned to assess whether intervention characteristics might have affected the differential effectiveness of behavioural weight management interventions, such as being digitally versus in-person delivered or number of intervention sessions, but there was limited variation in intervention characteristics across the included trials. We decided against conducting such analyses as it is unlikely they would be sufficiently powered.

All data were analysed using Stata V.17/SE (StataCorp. 2021. Stata Statistical Software: Release 17/SE).

### Role of the funding source

The funder of the study had no role in study design, data collection, data analysis, data interpretation or writing of the report.

### Patient and public involvement

The study concept was discussed with a patient representative; however, they did not formally feed into the study design or conduct.

## Results

**Figure 1:**
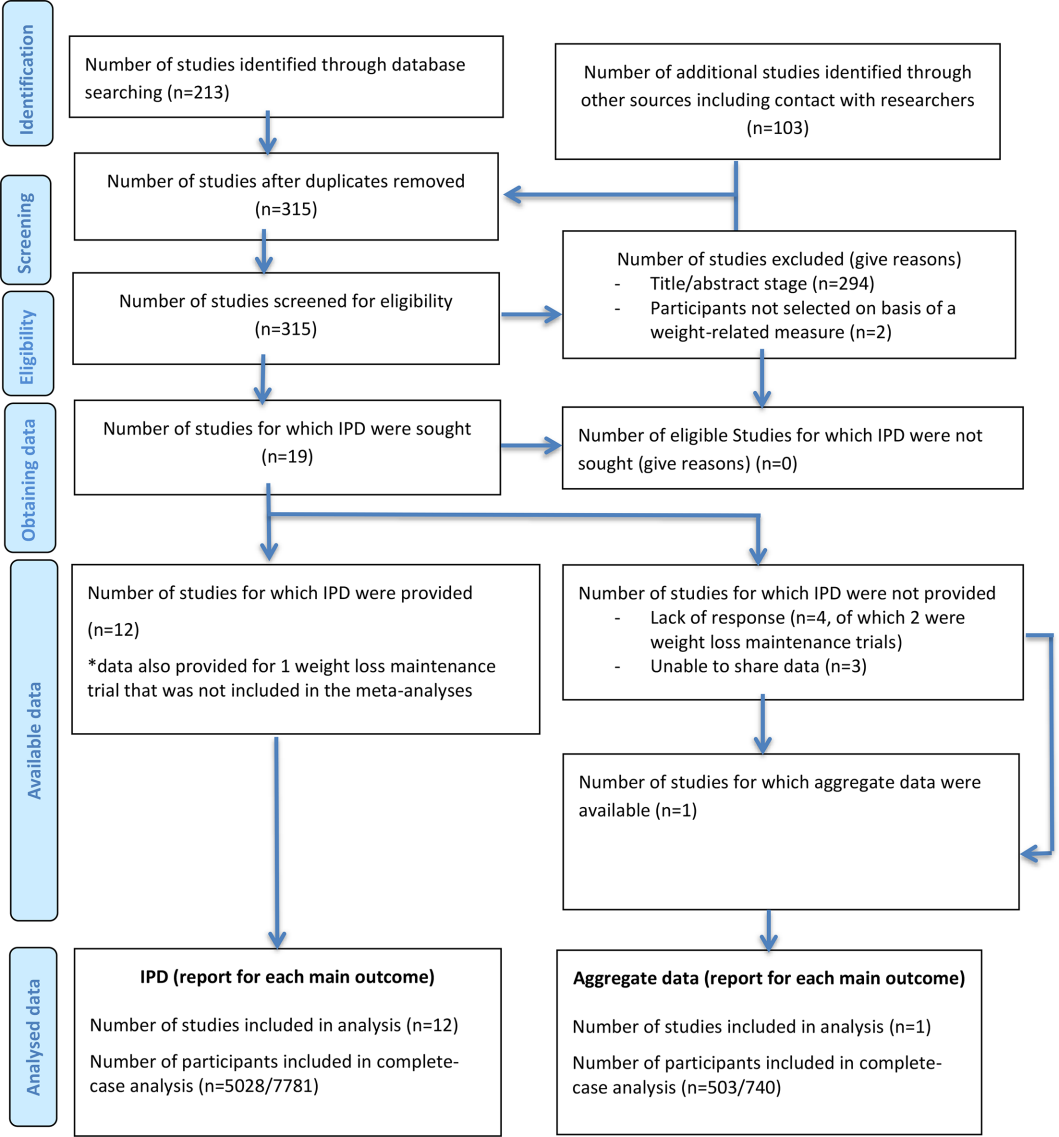
PRISMA-IPD flow chart. PRISMA-IPD, Preferred Reporting Items for a Systematic Review and Meta-analysis of Individual Participant Data. *As it was not possible to conduct a meta-analysis on 1 weight loss maintenance study we retrieved data for, we excluded this study from our analyses.

We identified 17 trials from our previous systematic review that met the inclusion criteria for this study.^18^,^34^ In the updated database search, we identified 212 unique records (shown in the PRISMA flow diagram,^35^ figure 1). Following title/abstract screening, we assessed four potentially eligible full-text articles and identified two further trials that met inclusion criteria.^36 37^ This led to 19 eligible trials for the IPD-MA in total. 16 of these were trials of behavioural weight loss interventions and 3 were trials of behavioural weight loss maintenance interventions (online supplemental table 2). We acquired and verified individual-level data for 12 out of 16 eligible weight loss trials (online supplemental table 3).^18^,^2123^ We were unable to access the data or obtain results of the analysis for three weight loss trials meeting the inclusion criteria.^22 28 37^ A collaborator was able to conduct the analyses and report the results for inclusion in meta-analysis in one further trial.^26^ We were only able to access the individual-level data for one weight loss maintenance trial, and it would have been inappropriate to meta-analyse this study with the weight loss trials, so we focused the IPD-MA on the weight loss trials.

### Study characteristics

Details of the data available from each trial are summarised in table 1. Across all the trials included in the analyses, there were 8521 participants, of which 94% of participants were of a white ethnicity, 57% of participants were female, the mean age was 54 years and 28% were from the least and 11% from the most deprived quintiles based on the Index of Multiple Deprivation (IMD). Online supplemental table 4 outlines how each PROGRESS-Plus characteristic was captured across the 13 included trials and the coding used for each characteristic in the IPD-MA. Complete case data were available for 5531 participants. Those of a white ethnicity and older participants had greater odds of having complete case data available; there was no evidence of attrition by area-level deprivation or gender. Further information on the demographics of the 5531 participants with complete case data is presented in online supplemental table 4.

**Table 1:** Characteristics of eligible (n=19) and included (n=13) studies

Study characteristic	Number (%)
Intervention type	
Weight loss	16 (84)
Weight loss maintenance	3 (16)
Country	
UK	19 (100)
Individual participant data available	
Weight loss studies	12 (63)
Weight loss maintenance studies	0 (0)
Analyses conducted by collaborator (individual participant data not accessible)	
Weight loss	1 (5)
Weight loss maintenance	0 (0)
Data available	
Weight loss	13 (100)
Weight loss maintenance	0 (0)
Weight change outcome	13 (100)
Attendance	5 (38)
Place of residence	1 (8)
Ethnicity	12 (92)*
Occupation	6 (46)
Gender/sex	13 (100)†
Religion	0 (0)
Education	8 (62)
Social capital (relationship status)	2 (15)
Socioeconomic status (IMD and Scottish IMD)	11 (85)‡
Socioeconomic status (annual household income)	2 (15)
Age	13 (100)‡

* Four of the 12 studies were not included in the ethnicity analyses as they either had only white participants, or too few participants from minority ethnic groups to conduct the regression analyses.

† Two studies (Football Fans in Training and ActWELL) were targeted towards participants of a single sex and are excluded from the gender/sex analyses.

‡ One study included in this table (10TT) coded SES and age differently from other studies, and it was not possible to harmonise these variables and include 10TT in the meta-analyses for these characteristics.

IMD, Index of Multiple Deprivation; SES, socioeconomic status.

Most trials in the IPD-MA had at least two in-person sessions as part of the intervention.^18^,^2023^ Nine interventions were delivered primarily in person,^1820 23^,^26 29 30 32^ three primarily through remote methods such as through phone calls, emails and web-based sessions^19 27 36^ and one was solely a self-guided leaflet-based intervention.^21^ Overall, we found that being in the intervention group was associated with 2.38 kg lower weight at 12-month follow-up than the control group (95% CI 1.43 to 3.33; p<0.001; I^2^=86%; tau^2^=2.47). This ranged from 0.06 kg lower weight (95% CI −1.13 to 1.26) in the 10TT trial^21^ to 7.31 kg (95% CI 4.93 to 9.70) in the DROPLET trial.^32^

### Inequalities in intervention effectiveness

**Figure 2:**
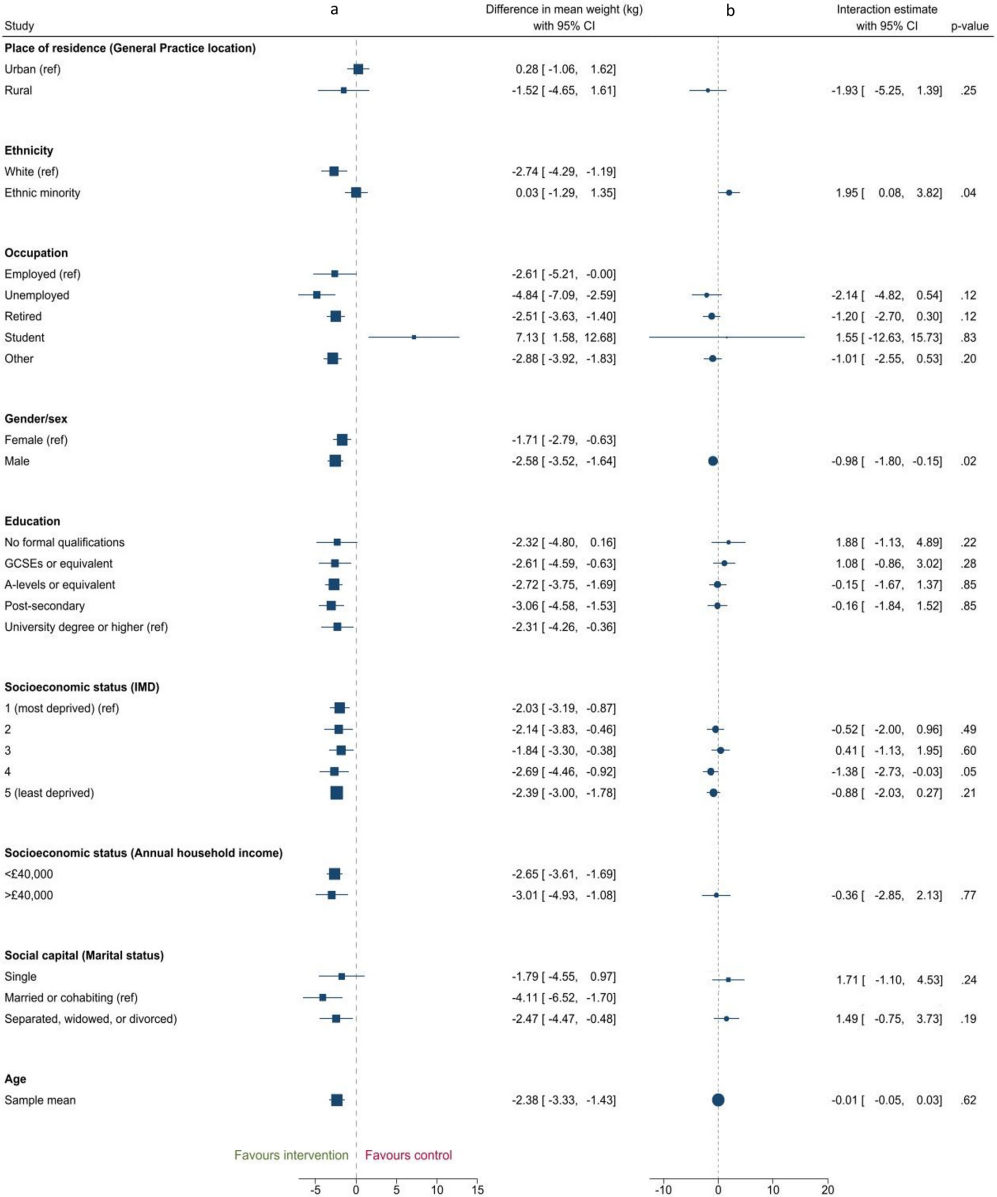
Forest plots showing: (a) Stratified analyses showing difference in mean weight at 12 months in the intervention group minus control for each characteristic subgroup and (b) Estimated interaction coefficients (difference in mean weight differences at 12 months) between intervention and inequality characteristic. GCSEs, General Certificate of Secondary Education; IMD, Index of Multiple Deprivation.

There was evidence of interactions between intervention and gender/sex and between intervention and ethnicity (figure 2). The between-group difference in mean weight at 12 months was greater in males (−2.58 kg (95% CI −3.52 to 1.64),) than females (−1.71 kg (95% CI −2.79 to –0.63)), p value for interaction=0.02. Tau^2^, assessing between-studies variance in the interaction estimates, was 0 (online supplemental figure 1), and the 95% PI −1.94 to −0.02 (table 2). The proportion of total variability in the observed interaction effects due to heterogeneity (I^2^) was 0%. The difference in weight at 12 months was also greater in those of a white ethnicity (−2.74 kg (95% CI −4.30 to –1.19)) than those from an ethnic minority background (0.03 kg (95% CI −1.29 to 1.35), p value for interaction=0.04, tau^2^=0, I^2^=0%, 95% PI −0.52 to 4.52) (online supplemental figure 2).

**Table 2:** Estimated interaction parameters from random effects meta-analysis

Characteristic	Estimated interaction (95% CI)	I^2^	Tau^2^ (95% prediction interval of estimated interaction)	P value
Place of residence (general practice location) n=1 study				
Rural (vs urban)	−1.93 (-5.25 to 1.39)	n/a*	n/a*	0.25
Ethnicity n=8 studies				
Ethnic minority (vs white (including white minorities))	1.95 (0.08 to 3.82)	0%	0.00 (-0.38 to 4.28)	0.04
Occupation n=6 studies				
Unemployed (vs employed)	−2.14 (-4.82 to 0.53)	0%	0.00 (-6.49 to 2.21)	0.12
Retired (vs employed)	−1.20 (-2.70 to 0.30)	0%	0.00 (-4.49 to 2.09)	0.12
Student (vs employed)	1.55 (-12.63 to 15.73)	79%	163.76 (-61.70 to 64.80)	0.83
Other (vs employed)	−1.01 (-2.55 to 0.53)	0%	0.00 (-3.51 to 1.49)	0.20
Gender/sex n=11 studies				
Male (vs female)	−0.98 (-1.80 to 0.15)	0%	0.00 (−1.94 to –0.02)	0.02
Education n=8 studies				
Postsecondary (vs university)	−0.16 (-1.84 to 1.53)	21%	0.79 (-4.10 to 3.78)	0.85
A-Levels/equivalent (vs university)	−0.15 (-1.67 to 1.37)	8.23%	0.32 (-2.82 to 2.52)	0.85
GCSEs/equivalent (vs university)	1.08 (-0.86 to 3.02)	0.00%	0.00 (-3.18 to 5.34)	0.28
No formal qualifications (vs university)	1.88 (-1.13 to 4.89)	2.45%	0.21 (-18.48 to 22.24)	0.22
Socioeconomic status (IMD or Scottish IMD) n=10 studies				
IMD quintile 2 vs 1 (most deprived)	−0.52 (-2.00 to 0.95)	0.00%	0.00 (-2.31 to 1.27)	0.49
IMD quintile 3 vs 1	0.41 (-1.13 to 1.95)	12.17%	0.67 (-2.27 to 3.09)	0.60
IMD quintile 4 vs 1	−1.38 (-2.73 to 0.03)	0.00%	0.00 (-3.01 to 0.25)	0.05
IMD quintile 5 (least deprived) vs 1	−0.88 (-2.03 to 0.27)	0.00%	0.00 (-2.59 to 0.83)	0.21
Socioeconomic status (annual household income) n=2 studies				
>£40 000 (vs <£40 000)	−0.36 (-2.86 to 2.13)	31%	1.04 (n/a†)	0.77
Social capital n=2 studies				
Single (vs married/cohabiting)	1.71 (-1.11 to 4.52)	0%	0.00 (n/a†)	0.24
Separated/widowed/divorced (vs married/cohabiting)	1.49 (-0.75 to 3.72)	0%	0.00 (n/a†)	0.19
Age n=11 studies				
Per 1 year of age at baseline	−0.01 (-0.05 to 0.03)	20%	0.00 (-0.06 to 0.04)	0.62

The interaction estimates are the difference in the effect of the intervention (vs control) comparing the listed 2 categories of each characteristic or per year of age.

* As there was only one study that reported data on place of residence, the estimated interaction comes from one study and therefore no meta-analysis was conducted

† As there were two studies in this analysis, it was not possible to calculate a prediction interval

GCSEs, General Certificates of Secondary Education; IMD, Index of Multiple Deprivation.

The intervention had a greater effect in those in the fourth least deprived IMD quintile compared with the most deprived quintile (−1.38 kg, 95% CI −2.73 to –0.03, p=0.05), although there was no evidence of this when comparing the most and least deprived quintiles (−0.88 kg, 95% CI −2.25 to 0.49, p=0.21). There was no evidence of a difference in mean weight change between intervention and control by place of residence, occupation, education, socioeconomic status (quintiles 5, 3 and 2 vs 1), socioeconomic status (annual household income), social capital (marital status) and age (table 2).

We conducted post hoc analyses for gender/sex and ethnicity to understand how these interactions occurred (eg, to understand whether the observed interaction effects could be particularly attributable to differences in the intervention or control groups alone). To understand the interaction between gender/sex and intervention, we examined differences in weight between males and females within each randomised group. We found that weight at follow-up controlled for baseline weight, was 1.27 kg (95% CI 0.09 to 2.45) higher in males than females in the control group and 0.09 kg (95% CI −0.65 to 0.83) higher in males than females in the intervention group. For ethnicity, those from an ethnic minority background had a 0.10 kg (95% CI −1.03 to 0.82) lower weight at follow-up than white participants in the control group and a 0.94 kg (95% CI −0.94 to 2.84) higher weight at follow-up than white participants in the intervention group.

### Inequalities in intervention attendance

We were able to harmonise data on intervention attendance from five trials (online supplemental table 5).^1823^,^26^ The number of core available sessions in each trial ranged from 9 in ‘Waste the Waist’^23^ to 52 in WRAP and Jebb 2011.^18 25^ We did not find evidence of inequalities in intervention attendance by ethnicity, occupation, gender/sex, socioeconomic status (IMD) or age. We did find some evidence of differential trial attendance by socioeconomic status (annual household income), but the difference was small (ie, less than one session) and the data came from a single study.

### Cohort analyses

In cohort analyses, we found evidence of associations of gender/sex, social capital (marital status) and age, with weight at follow-up (online supplemental table 6). Being male was associated with a higher weight at 12-month follow-up (adjusted for baseline) of 0.62 kg (95% CI 0.10 to 1.15; p=0.02; I^2^=27%; tau^2^=0.20) compared with being female. Being separated, widowed or divorced was associated with having a higher weight of 1.31 kg (95% CI 0.18 to 2.43) at follow-up versus those who were married or cohabiting. Older age at baseline was associated with a lower weight at follow-up; a 10-year increase in age at baseline was associated with a lower weight of 0.5 kg (95% CI 0.4 to 0.7; p<0.001; I^2^=0.01%; tau^2^=0.00). There did not appear to be an association between follow-up weight and place of residence, ethnicity, occupation, education or socioeconomic status (IMD and annual household income).

### Risk of bias

**Figure 3:**
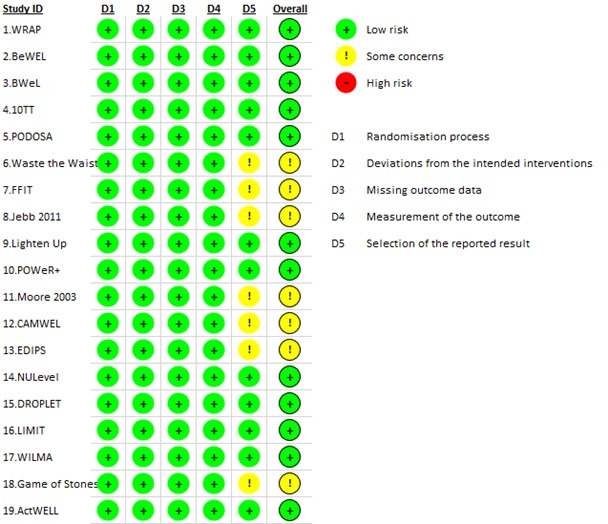
Risk of bias assessment using Cochrane’s RoB 2 tool.

None of the 19 eligible studies were assessed to be of a high risk of bias using Cochrane’s RoB 2 tool (figure 3). We determined 12 to be of low risk of bias,^18^,^2226 27 31^ and 7 to have some concerns of risk of bias.^23^,^2528^ All seven studies scoring some concerns of risk of bias did so because of not having a retrievable prespecified analysis plan available. In the sensitivity analyses, removing the trial that did not provide individual-level data did not alter the observed associations between exposures and outcomes.

## Discussion

In this IPD-MA, we analysed data from 13 RCTs of behavioural weight loss interventions. We found that there was a stronger intervention effect in people of white ethnicity than in those from a minority ethnic background. We also found that these interventions also had a stronger effect in men than women, but that this effect was driven by differences in weight change in the control group rather than the intervention group. We found some evidence of differential intervention effectiveness by area-level deprivation, but this was not consistent. We did not find evidence of differential intervention effectiveness by other characteristics including age, education, occupation and household income. We did not find evidence of inequalities in intervention attendance by ethnicity, occupation, gender/sex, education, area-level socioeconomic deprivation or age.

The English NHS Diabetes Prevention Programme provides a useful healthcare-based comparison to the results of our study, as those interventions are similar to most interventions included in this study, in terms of content, modality and duration. The NHS DPP observed lower weight loss in people of female sex, Asian and black ethnicity, lower socioeconomic status and younger age.^38^ The findings are similar even though there are differences in the respective populations and trial versus non-trial study contexts. The English NHS Diabetes Prevention Programme enrols a higher proportion of men than these behavioural weight management interventions, where referral for intervention and enrolment in behavioural weight management trials is more likely among women than men.^39 40^

Our finding that behavioural weight management interventions are more effective in those of a white ethnicity, despite no evidence of differences in attendance, suggests that the inequalities in the weight outcomes are likely being generated by the intervention itself. Our previous narrative systematic review found some evidence that the effectiveness of behavioural weight management interventions favoured those of a white ethnicity, although most trials were not sufficiently powered to explore differential effectiveness.^11^ Fitzgibbon *et al* also found that black women had lower weight loss than other ethnicities.^41^ Qualitative research has previously explored how engagement with, and adherence to, an intervention differs by cultural context and found that the general tailoring of behavioural weight management interventions towards white British participants can present barriers preventing the implementation of intervention content in daily life for participants with different ethnic backgrounds.^42 43^ For example, many behavioural weight management interventions offer dietary advice that is not transferable to typical food preparation methods used in some cultures, or affordable for people on low incomes to implement.^42^ Similarly, interventions that use physical activity provision as a component and do not contain access to single-sex spaces to exercise may prevent those of certain cultural backgrounds—such as British Pakistani Muslim women—from accessing such provision.^43^ Future research should investigate the mechanisms behind the observed inequalities by ethnicity so that people with obesity from minority ethnic groups can be better supported to manage their weight in ways that are culturally appropriate. This research should also explore for which minority ethnic groups in particular behavioural weight management interventions do not work for, so they can be equitably supported in managing their weight.

Our finding that the intervention effect was stronger in those of male sex is similar to observations from NHS DPP that those of female sex lost less weight and another meta-analysis also found the effectiveness of weight loss interventions favoured men, although the difference was small.^44^ However, our post hoc analyses suggested the greater treatment effect observed in males may, in part, have been produced by men doing worse in the control group than women despite having comparable follow-up weight in the intervention group. This suggests that when men are provided with a minimal intervention, they may have greater difficulty in managing their weight than women. The comparable outcomes in the treatment group between males and females suggest that despite typically lower uptake of these interventions by men, they achieve similar outcomes to women and that future work should consider how to achieve greater utilisation of these general weight management interventions by men. These generic interventions are particularly useful—especially where gender-tailored weight management programmes towards men (such as Football Fans in Training and Man v Fat Football) may not be available or appeal to some men.^24 45 46^

Although we found some evidence that behavioural weight management interventions were more effective in those from less deprived groups, this evidence was not consistent across the socioeconomic gradient (ie, lesser intervention effectiveness compared with the least deprived group was noted in the fourth most but not the most deprived quintiles). This broadly compares to the findings of previous systematic reviews, which found that individual-level and primary care delivered interventions are unlikely to increase inequalities in obesity.^47 48^ We did not find evidence of inequalities in attendance, which is comparable to previous literature that found despite greater trial attrition in disadvantaged groups, there was no evidence of differential intervention adherence.^41^ Similarly, the rate of trial attrition being higher in those from an ethnic minority background supports the findings from Fitzgibbon and colleagues in the USA.^41^

To the best of our knowledge, this is the first IPD-MA to consider inequalities in the attendance and effectiveness of behavioural weight management interventions. Accessing individual-level data allowed for harmonisation (recoding variables across trials so they were consistently coded) of the PROGRESS-Plus characteristics and attendance data, which allowed us to include data from more trials in the meta-analysis than would be possible using aggregate data from study publications. This highlights the usefulness of IPD-MA in considering differential effectiveness of interventions, due to the increased statistical power from the harmonisation and synthesis of the PROGRESS-Plus-related data.^49^ The IPD-MA approach provides a more precise answer on the extent of inequalities in behavioural weight management interventions, especially when compared with previous systematic reviews that used narrative synthesis approaches when considering inequalities in intervention attendance or effectiveness.^5 11 48 50 51^

There are some limitations to this study and the trials included. First, our search included studies published up to 31 December 2021. While this time from search to publication is typical for IPD-MA studies (which often take from 2 to 4 years to be published since the search was completed),^52^,^54^ it may have led to more contemporary studies not being included. To the best of our knowledge, only one such study would have been missed—the Game of Stones trial published in 2024.^55^ Second, it was not possible to harmonise all data across the PROGRESS-Plus characteristics that we received. For example, age and IMD data for the 10TT trial was received in 10-year increments and tertiles, respectively.^21^ We decided to exclude 10TT from the meta-analyses of age and IMD to preserve the detail of the remaining studies which provided age as a continuous outcome and IMD in quintiles. Similarly, we considered ethnicity as binary (white (including white minorities) vs ethnic minority), as this is how the data in several of the included trials was shared. In part, this was because of small numbers within each group. Likewise, of the three studies we were not able to access data or results for, two (Dombrowski *et al*^37^ and Bhopal *et al*^22^) had a relatively small number of participants (n=105 and 171, respectively). Given that each of these studies focused on a single characteristic (men and those of South Asian origin), neither of these studies would have contributed to the interaction analyses for gender/sex and ethnicity. Therefore, it is unlikely that inclusion of these studies in particular would have added statistical power to our analyses. Although the third study (Moore *et al*^28^) had a good number of participants (n=843), the baseline characteristics table in the original paper suggests this study would have only contributed to our gender/sex and age analyses. Another limitation of the Moore *et al*’s^28^ study is that the intervention is of a health practitioner education programme, rather than one delivered to the participants. Future research should ensure greater diversity in the study population, so that greater consideration can be given to specific ethnic groups these interventions are currently ineffective for, and why that may be the case.

Third, the estimates of inequality are influenced by the distribution of characteristics within each study. For example, studies with a narrow age range might not identify interactions between intervention effects and age. This may be indicative of the lack of diversity and low inclusion of ‘underserved groups’ in trial populations more generally,^56 57^ where participants are often more likely to be White and more affluent than the general population. Additionally, the lack of inequalities observed across characteristics considered in this study—such as by occupation, education and annual household income—may indicate that inequalities are present either among trial participants or in intervention uptake, which we were not able to consider in this study. As all trials included in this study were UK-based, the generalisability of the findings to other countries or healthcare settings may be limited. Finally, there were several PROGRESS-Plus characteristics for which minimal or no data were available (such as place of residence and sexual orientation); future trials may wish to use the PROGRESS-Plus framework when planning data collection relating to baseline characteristics to facilitate analyses of the full range of characteristics that may lead to health inequalities. Related, as is typical in behavioural weight management trials, there was a high amount of missing data (~35%).^58^ This includes information on intervention attendance, which we were only able to assess across five trials. We did not consider intersectionality between different characteristics where inequalities occur in this study. It is, however, an important issue for future research to address intersectionality in terms of differential intervention outcomes, as well as building on recent research that explored intersectional differences in prevalence of obesity.^59^,^61^

In this first IPD-MA of inequalities in behavioural weight management interventions, we have shown that behavioural weight management interventions are likely to be less effective for people from an ethnic minority background. We did not find evidence of differential intervention attendance in the subsection of studies we had attendance data available for. Future research should investigate the mechanisms behind this, such as exploring how behavioural weight management interventions may be culturally tailored, to better support people with obesity from minority ethnic groups. Furthermore, given the positive intervention effect observed in men, future work should consider how to increase the uptake of behavioural weight management among men, given the typically lower utilisation of these interventions by men in practice.

## Supplementary material

10.1136/bmjph-2024-001382 online supplemental file 1

## Data Availability

Data may be obtained from a third party and are not publicly available.
