# Supplementary material for: Are there inequalities in the attendance at and effectiveness of behavioural weight management interventions for adults in the UK? An individual participant data meta-analysis
Source: BMJ Public Health. 2025 Aug 7;3(2):e001382. doi: 10.1136/bmjph-2024-001382 (PMC12336588; doi:10.1136/bmjph-2024-001382)
Supplement: online supplemental file 1 [file bmjph-3-2-s001.docx]

Supplementary material

[Supplementary Table 1 Medline search strategy 2](#_Toc197692211)

[Supplementary Table 2 Summary of eligible studies for inclusion in individual participant data meta-analysis 4](#_Toc197692212)

[Supplementary Table 3 Capturing of PROGRESS-Plus characteristics in each individual trial and overall harmonised variables 12](#_Toc197692213)

[Supplementary Table 4 Summary of participants included in complete case analyses where individual participant data were accessed 19](#_Toc197692214)

[Supplementary Table 5 Percentage difference between groups for intervention attendance 20](#_Toc197692215)

[Supplementary Table 6 Difference in weight at 12-months across the cohort of participants in trials of behavioural weight management interventions 21](#_Toc197692216)

[Supplementary Figure 1 Forest plot of the difference (male minus female) in mean differences between the intervention and control groups for gender/sex 22](#_Toc197692217)

[Supplementary Figure 2 Forest plot of the difference (White minus Ethnic Minority) in mean differences between the intervention and control groups for ethnicity 22](#_Toc197692218)

# Supplementary Table 1 Medline search strategy

| 1 Obesity/  2 Obesity, Morbid/  3 Overweight/  4 Obesity, Metabolically Benign/  5 Weight loss/  6 obes$.ti.  7 overweight.ti.  8 weight.ti.  9 (adipos$ or body fat).ti.  10 (obes$ or overweight or weight loss).ti,ab.  11 limit 10 to ("in data review" or in process or "pubmed not medline")  12 1 or 2 or 3 or 4 or 5 or 6 or 7 or 8 or 9 or 11  13 Weight Reduction Programs/  14 Behavior Therapy/  15 Cognitive Therapy/  16 Counseling/  17 Directive Counseling/  18 Self-Help Groups/  19 counsel$.ti,ab.  20 (behav$ adj3 (therap$ or program$ or intervention$)).ti,ab.  21 Health Education/  22 Diet, Reducing/  23 Diet, Fat-Restricted/  24 Caloric Restriction/  25 Diet Therapy/  26 (diet$ adj counsel$).ti,ab.  27 (diet$ adj education$).ti,ab.  28 (nutrition$ adj counsel$).ti,ab.  29 (nutrition$ adj education$).ti,ab.  30 (nutrition$ adj intervention$).ti,ab.  31 (diet$ adj (modif$ or therapy or intervention$ or strateg$)).ti,ab.  32 ((diet or dieting or slim$) adj (club$ or organi?ation$)).ti,ab.  33 (weight reduc$ adj diet$).ti,ab.  34 (weightwatcher$ or weight watcher$).ti,ab.  35 Exercise/  36 Exercise Therapy/  37 Motor Activity/  38 Physical Conditioning, Human/  39 Physical Fitness/  40 physical activity.ti,ab.  41 (exercise adj3 (therap$ or program$ or intervention$)).ti,ab.  42 ((lifestyle or life style) adj (modification$ or intervention$)).ti,ab.  43 13 or 14 or 15 or 16 or 17 or 18 or 19 or 20 or 21 or 22 or 23 or 24 or 25 or 26 or 27 or 28 or  29 or 30 or 31 or 32 or 33 or 34 or 35 or 36 or 37 or 38 or 39 or 40 or 41 or 42  44 12 and 43  45 Obesity/dh, th, dt, rh [Diet Therapy, Therapy, Drug Therapy, Rehabilitation]  46 Obesity, Morbid/dh, th, dt, rh  47 Overweight/dh, th, dt, rh  48 (weight loss adj (intervention$ or program$ or trial$)).ti,ab.  49 (weight reduc$ adj (intervention$ or program$ or trial$)).ti,ab.  50 (weight management adj (intervention$ or program$ or trial$)).ti,ab.  Limits:   - Studies published between 5^th^ March 2020 and 31^st^ December 2021 - United Kingdom-based studies only |
| --- |

# Supplementary Table 2 Summary of eligible studies for inclusion in individual participant data meta-analysis

| Study name  (first author) | Year published | Intervention group description | Control group description | Participant eligibility criteria |
| --- | --- | --- | --- | --- |
| WRAP (Ahern) | 2017 | Participants were given vouchers to attend Weight Watchers (WW) meetings once a week and access WW digital tools for the duration of their intervention (12- or 52-weeks) for free. | A printed booklet of self-help weight-management strategies (British Heart Foundation). | Inclusion: Aged >18 years and BMI >28 kg/m^2^.  Exclusion: Planned or current pregnancy, previous or planned bariatric surgery, current participation in a weight-loss programme, having an eating disorder, non-English speaking. |
| BeWEL (Anderson) | 2014 | 12-month intervention delivered by trained lifestyle counsellors in 3 x 1-hour one-to-one visits during the first 3 months, followed by 9 monthly 15-minute telephone conversations, leading to a total contact time of 5.25 hours. | A printed booklet of self-help weight-management strategies (British Heart Foundation). | Inclusion: Aged 50 to 74 years, had undergone polypectomy for adenoma, and BMI >25 kg/m^2^.  Exclusion: Pregnancy, insulin dependent diabetes mellitus, and any cancer diagnosis. |
| BWeL (Aveyard) | 2016 | General Practitioners offered participants referral to a commercial weight management programme of 12 1-hour sessions (Slimming World, Rosemary Conley) and gave vouchers to allow them to attend for free. | General Practitioners advised participants to lose weight | Inclusion: Aged > 18 years, BMI >25 kg/m^2^ (if Asian ethnicity) or BMI >30 kg/m^2^ (if other ethnicities), have a raised body fat percentage.  Exclusion: Planned or current pregnancy, previous bariatric surgery, completed or participating in a weight management programme within previous 3 months, non-English speaking. |
| Ten Top Tips *[10TT]* (Beeken) | 2017 | 10TT was a self-guided leaflet-based intervention that used habit-formation theory to aid weight loss. A logbook was provided for participants to self-monitor target behaviours. | Usual care, dependent on the participant’s General Practitioner. May include dietary advice or referral to a commercial programme. | Inclusion: Aged > 18 years, BMI > 30 kg/m^2^.  Exclusion: Unable to provide informed consent due to mental incapacity or active psychotic illness, pregnant, or terminally ill. |
| PODOSA (Bhopal) | 2014 | 15 visits from a dietitian over 3 years, where the dietitian would advise participants on achieving weight loss through culturally adapted and translated resources. | 4 visits from a dietitian over 3 years where standard advice on healthy eating, diabetes prevention and physical activity was given. | Inclusion: Aged > 35 years, self-identified men and women of Indian or Pakistani origin with waists measuring > 90cm (men) or > 80cm (women), impaired glucose tolerance or impaired fasting glucose tolerance, the family cook was cooperative.  Exclusion: Receiving long-term oral corticosteroids or weight loss medication, having long-term health disorders making adherence improbable, pregnant, and unlikely to remain in the UK for 3 years. |
| Waste the Waist (Greaves) | 2015 | 4 x 2-hour group-based sessions in the first month to support behaviour change for weight loss, then 5 x 90-minute group sessions over the next 8 months to support maintenance of behaviour change, totalling 13.5 hours of contact time. | Participants were provided written information on the effects of diet and physical activity on cardiovascular risk. | Inclusion: Aged 40-74 years, BMI > 28 kg/m^2^, and having a high cardiovascular risk defined using either the Framingham or QRISK2 algorithm.  Exclusion: Existing heart disease, type 2 diabetes mellitus, BMI > 40 kg/m^2^, |
| Football Fans in Training (Hunt) | 2014 | 12 weekly sessions of 90-minutes in length, delivered at 13 Scottish professional football club stadiums. Each 90 min session combined advice on healthy diet with physical activity. The balance of classroom and physical activity sessions changed during the 12 weeks; later weeks focused on physical activity as men became fitter, and the shorter classroom sessions focused on revision. The 12-week active phase was followed by a weight maintenance phase with six post-programme email prompts during 9 months and a group reunion at the club 6 months after the end of the sessions. | 12 month waiting list to receive the FFIT intervention. | Inclusion: Men, aged 35-65 years, BMI > 28 kg/m^2^, completed physical activity readiness questionnaire, not taken part in FFIT previously.  Exclusion: Blood pressure that contraindicated vigorous exercise (systolic ≥160 mm Hg or diastolic ≥100 mm Hg) were excluded from the more intense physical activity programme sessions. |
| (Jebb) | 2011 | Participants were given vouchers to attend WW (previously Weight Watchers) meetings once a week for 12 months and access digital tools for free. | Advice from their GP and other standard care in line with national treatment guidelines. | Inclusion: Aged > 18 years, BMI 27-35 kg/m^2^, and at least one risk factor for obesity related disease (such central adiposity, type 2 diabetes mellitus without inulin treatment, family history of diabetes).  Exclusion: Achieved weight loss of > 5kg in previous 3 months, history of clinically diagnosed eating disorder, orthopaedic limitations preventing regular physical activity, untreated thyroid disease or more than one change in thyroid treatment in the previous 6 months; receiving treatment with effects on weight or appetite; gastrointestinal disorders; previous surgical procedure for weight loss; major surgery in the previous 3 months; pregnancy or lactation; insulin-treated diabetes; diabetes diagnosis in the previous 6 months; glycated haemoglobin (HbA1c) of at least 75 mmol/mol (9·0%); heart problems in the previous 3 months; uncontrolled hypertension; new prescription drug for a chronic disorder in the previous 3 months or change in dose in the previous 1 month; history or presence of cancer, with the exception of completely resected basal or squamous cell carcinoma if treatment completed 6 months before enrolment or if treatment was stable; or participation in another clinical trial in the previous 30 days. |
| Lighten Up (Jolly) | 2011 | In addition to 12 vouchers for free entrance to a local leisure centre, participants were randomised to one of 7 intervention groups (all 12 weeks in length): Weight Watchers, Slimming World, Rosemary Conley, Size Down, GP-led one-to-one counselling, pharmacy-led one-to-one counselling, or a choice of any of the six intervention programmes. | 12 vouchers for free entrance to a local leisure centre. | Inclusion: Registered with general practices in the South Birmingham Primary Care Trust, aged > 18 years, had a raised BMI recorded in primary care notes within previous 15 months (White Europeans and all ethnic groups apart from South Asians with no comorbidities BMI 30 > kg/m^2^, White Europeans and all ethnic groups apart from South Asians with comorbidities BMI 28 > kg/m^2^, South Asians with no comorbidities 25 > kg/m^2^, South Asians with comorbidities 23 > kg/m^2^).  Exclusion: Unable to understand English or were pregnant. |
| POWeR+ (Little) | 2016 | Two intervention groups, both consisting of a 24-session web-based weight management programme lasting 6 months.  POWeR+F provided three scheduled (and four optional) face-to-face nurse support sessions.  POWeR+R included three phone or email contacts and two optional phone or email contacts. | Brief advice web-pages for a healthier diet. | Inclusion: Aged > 18 years and BMI > 30 kg/m2 or more (or ≥ 28 kg/m2 with hypertension, hypercholesterolaemia, or diabetes).  Exclusion: Severe mental health problems, too ill to participate in the study or unable to change diet due to health, pregnant or breastfeeding, perceived inability to walk 100m, another member of household participating, no regular access to the internet. |
| (Moore) | 2003 | Intervention was targeted at general practitioners and practice nurses, and the unit of randomisation was primary care practice. The intervention consisted of 3x90-minute sessions that trained the practitioners on a model approach to obesity treatment. | Control practices were asked to provide usual care to patients. | Inclusion: Aged 16-64 years and BMI > 30 kg/m^2^. |
| CAMWEL (Nanchahal) | 2012 | One-to-one programme delivered across 14 visits over 12 months by advisors trained in obesity causes, diet and physical activity, behaviour change strategies, motivational interviewing and cognitive behavioural therapy techniques. | Usual care. | Inclusion: Aged > 18 years and BMI >25 kg/m^2^. |
| EDIPS (Penn) | 2009 | Participants received regular individual advice from a dietitian and physiotherapist trained in motivational interviewing. Participants were also invited to group events, such as ‘cook and eat’. Individual sessions were for 30 minutes monthly for the first 3 months and then every 3 months for up to 5 years. | Brief advice and usual care from GP. | Inclusion: Aged >40 years, BMI > 25kg/m^2^, and impaired glucose tolerance of ≥ 7.8 mmol/l and < 11.1 mmol/l.  Exclusion: Previous diagnosis of diabetes mellitus, chronic illness that makes participation in moderate physical activity impossible, or on a special diet for medical reasons. |
| DROPLET (Astbury) | 2018 | The intervention was a Total Diet Replacement programme, which consisted of weekly behavioural support for 12 weeks and monthly support for 3 months with formula food products providing 810 kcal per day for the first 8 weeks, followed by gradual reintroduction of food. | Behavioural support for weight loss from a practice nurse and a diet programme with modest energy restriction. | Inclusion: Aged > 18 years, BMI *>* 30kg/m^2^, and participants’ GP determines weight loss would benefit health.  Exclusion: Scheduled or previously received bariatric surgery, currently participating in a weight management programme, and contraindications to total diet replacement. |
| Game of Stones feasibility (Dombrowski) | 2020 | Two intervention groups. Both intervention groups received narrative text messages for 12 months. The text messages were written from the perspective of a fictional character aiming to lose weight over 12 months and targeted towards men from disadvantaged backgrounds. Between 0 and 5 texts were sent a day over 12 months. In addition to this, one intervention group received financial incentives dependent on meeting weight loss targets. | Wait-list control | Inclusion: Men, aged > 18 years, BMI > 30kg/m^2^ and/or waist circumference of > 102cm, owned a mobile phone capable of receiving text messages, understand English, considered by practice clinical staff as suitable for participation (GP practice recruitment only), for example, no severe medical, terminal or psychiatric illness (in patient or close family member) or no significantly impaired cognitive function.  Exclusion: Taking part in a weight loss study, planning or waiting to have bariatric surgery, planning to move within 12 months from study baseline. |
| ActWELL (Anderson) | 2021 | Twelve-month long intervention based on COM-B model of behaviour change delivered by Breast Cancer Now volunteers. There were two one-to-one sessions in first 12-weeks of the intervention and 9 x 15-minute support calls over the following nine months. | Brief advice leaflet. | Inclusion: Attending, or invited to attend, routine breast screening clinics (not recall clinics), BMI > 25kg/m^2^, age 50 to 70 years.  Exclusion: Undergoing treatment for any malignant condition (excluding certain skin cancers), reported contra-indications to physical activity or weight loss, on a specialised medical diet, diagnosis of T1 diabetes, current use of insulin, no telephone contact, unable to consent. |
| NULevel (Sniehotta)  *Weight loss maintenance* | 2019 | Intervention was delivered via a combination of a single face-to face meeting and regular text messaged (at least 1 every 2 days). The text messages consisted of content that was triggered by participants daily self-weighing and questionnaire completion. | Brief lifestyle advice received by text message on 4 occasions, 3 months apart. | Inclusion: Aged > 18 years, BMI > 30kg/m^2^ (> 28kg/m^2^ if of South Asian descent) in the 24 months preceding trial, and had lost >5% of body weight in the preceding 12 months.  Exclusion: Lost weight through illness or surgical procedures, pregnant or planning to become pregnant during study period, breastfeeding, unable to understand English, diagnosis of an eating disorder or condition that limits physical activity, or plans to leave geographic area during study period. |
| LIMIT  (Daley)  *Weight loss maintenance* | 2019 | Over the course of a 12-week weight maintenance programme, participants carried out daily self-weighting, received 3 brief support phone calls delivered by non-specialist call centre staff, and text messages sent every other day for the first 4 weeks and twice weekly for the remaining 8 weeks. | Brief advice leaflet. | Inclusion: Aged > 18 years, had lost > 5% of their weight by the end of their weight loss programme, owned a mobile or landline phone that could receive text messages, was able to understand English sufficiently to complete study procedures.  Exclusion: Pregnant or intending to become pregnant during the study period. |
| WILMA (Simpson)  *Weight loss maintenance* | 2015 | Two intervention groups, both comprising of a 12-month intervention. Participants in both intervention groups could attend 4 peer group support sessions lasting 1.5 hours for 4 months following the face-to-face sessions.  Intensive group – participants received 6 one-to-one face-to-face individually tailored motivational interviewing delivered fortnightly for 3 months (each session listing around 60 minutes). In the remaining 9 months of the intervention, participants received monthly motivational interviewing calls lasting around 20 minutes.  Less intensive group – participants received two face-to-face motivational interviewing sessions two weeks apart and two motivational interviewing phone calls at 6 months and 12 months. | Brief advice leaflet. | Inclusion: Aged 18-70 years, current or previous BMI of > 30 kg/m^2^, and intentionally lost >5% of body weight in previous 12 months.  Exclusion: Previous bariatric surgery (unless reversed), terminal illness, inability to understand study materials in English, living with another study participant, or currently pregnant. |

# Supplementary Table 3 Capturing of PROGRESS-Plus characteristics in each individual trial and overall harmonised variables

| Study | Place of residence | Ethnicity | Occupation | Gender/ sex | Religion | Education | Social capital | Socioeconomic status | Plus |
| --- | --- | --- | --- | --- | --- | --- | --- | --- | --- |
| Ahern 2017  (WRAP) | - | 2011 UK census (White-British/White-Irish/White-Other/Chinese/Asian or Asian-British – Indian/Asian or Asian-British – Pakistani/Asian or Asian-British – Bangladeshi/ Asian or Asian-British – Other/Black or Black-British – Caribbean/Black or Black-British – African/Black or Black-British – Other/Mixed – White and Black Caribbean/Mixed – White and Black African/Mixed – White and Asian/Mixed – Other/Other/ Prefer not to say) | Employment status (Unemployed/Self-employed/ Employed by other/Student/ Retired/Unable to work/Other (carer, home-maker, voluntary work)/Prefer not to say) | Sex (Female/ Male) | - | (None/GCSEs or equivalent/ A-Levels or equivalent/Post secondary study/ University degree/Higher degree | Household member using weight loss programme (Yes/No) | English Indices of Multiple Deprivation [IMD] 2010 (continuous rank)  Household income (£0-9999 year/£10000-19999 year/£20000-29999/£30000-39999/£40000-49999/£50000-59999/£60000-69999/£70000+/ Don’t know/Prefer not to say) | Age (continuous) |
| Anderson 2014  (BeWEL) | - | 2001 UK census (White/Asian or Asian British/Chinese/Mixed/ Black or Black British/Other ethnic group/Do not wish to complete) | Employment status (Retired/Employed full time/Student full time/Unemployed/ Employed part time/Student part time/Unable to work/Other) | Sex (Female/ Male) | - | (Primary school/ Secondary school/Other professional or technical qualification after leaving school/ University degree/Post-graduate degree) | Marital status (Single/ Married or cohabiting/ Widowed or separated or divorced) | Scottish Index Multiple Deprivation [SIMD] 2012 (Quintiles)  Household Income (£280 week /£281-480 week/£481-770 week/£1250 month/£1251-2080 month/£2081-3330 month/<£15000 year/£15001-25000 year/£25001-40000 year/>£40000 year/prefer not to say) | Age (continuous) |
| Aveyard 2016  (BweL) | - | 2011 UK census (White/Black Caribbean/Black African/Mixed/Black other/Chinese/Indian/ Pakistani/Bangladeshi/ Other Asian/Other) | - | Gender (Female/ Male) | - | - | - | English Indices of Multiple Deprivation [IMD] 2010 (continuous rank) | Age (continuous) |
| Beeken 2017  (10TT) | Location of primary care physician (Urban vs rural) | Binary (White/ethnic minority) | - | Gender (Female/ Male) | - | - | - | English Indices of Multiple Deprivation [IMD] 2010 (terciles) | Age |
| Greaves 2015  (Waste the Waist) | - | All participants were White | - | Gender (Female/ Male) | - | (Up to age 16 or less/Up to age 18/Some additional/ Undergraduate degree or higher) | - | English Indices of Multiple Deprivation [IMD] 2000 (continuous rank) | Age (continuous) |
| Hunt 2014  (FFIT) | - | 2001 UK Census (White – British/White – Scottish/White – Irish/White – Any other white background/Mixed – White and Black Caribbean/Mixed – White and Black African/Mixed – White and Asian/Mixed – Any other Mixed background/Chinese/ Asian or Asian British – Indian/Asian or Asian British – Pakistani/Asian or Asian British – Bangladeshi/Asian or Asian British – Any other Asian background/Black or Black British – Caribbean/Black or Black British – African/ Black or Black British – Any other black background/Other ethnic group – Any other) | Employment status (In paid employment or self-employed (or temporarily away)/ Doing unpaid work for a business that you own, or that a relative owns/Waiting to take up paid work already obtained/On a Government scheme for employment training/Looking for paid work or a Government training scheme/Intending to look for work but prevented by temporary sickness or injury/Permanently unable to work because of long-term sickness or disability /Going to college or university full-time (including on holiday)/ Retired from paid work/Looking after home or family/Doing something else) | Gender (Male) | - | (No educational qualifications/ Standard grades, O grades, O levels, GCE or GCSEs/ Highers, advanced highers, A levels/ Vocational qualification (e.g. SVQ/SCOTVEC)/ HNC/HND/ Degree (e.g. BA, BSc)/ Post-graduate qualification (e.g. MSc, PhD)/Other | Marital status (Single (never married)/ Married/ Separated/ Widowed/ Divorced/ Living with someone as a couple (but not married)/ Other (including civil partnership)) | Scottish Index of Multiple Deprivation [SIMD] 2009 (quintiles) | Age (continuous) |
| Jebb 2011 | - | 2001 UK census (Asian or Asian British – Indian /Asian or Asian British – Other/Black or Black British – African/Black or Black British – Caribbean/Mixed – White & Black African/ Mixed – White & Black Caribbean/Other/White – British/White – Irish/ White – Other) | - | Sex (Female/ Male) | - | - | - | - | Age (continuous) |
| Jolly 2011  (Lighten Up) | - | 2001 UK census (White British or Irish/South Asian/Black British, Caribbean or African/Mixed and other) | Employment status (Full time student/ Home carer/ Intermediate/  Managerial or Professional/Never worked or long-term unemployed/ Retired/ Routine and manual / Sick or disabled | Sex (Female /Male) | - | - | - | IMD (continuous rank) | Age (continuous) |
| Little 2016  (POWeR+) | - | - | - | Sex (Female/ Male) | - | Age left education (continuous) | - | English Indices of Multiple Deprivation [IMD] 2010 (continuous rank) | Age (continuous) |
| Nanchahal 2012  (CAMWEL) | - | Non-UK census categories (African/Asian Other/Bangladeshi/ Black Other/Caribbean/ Chinese/Indian/Iran/ Mixed Other/Other/ Pakistani/White and Asian/White and Black African/White and Black Caribbean/Irish White/ White British/White Irish/White Other) | Employment status (Employed/ Unemployed) | Gender (Female/ Male) | - | Highest qualification obtained (None/GCSE or equivalent/A-level or equivalent/ Degree level or higher/Other) | - | English Indices of Multiple Deprivation [IMD] 2010 (quintiles) | Age (continuous) |
| Penn 2009  (EDIPS) | - | Binary (White/Ethnic minority) | - | Sex (Female/ Male) | - | Educational level (Low/Medium/ High) | - | - | Age (continuous) |
| Astbury 2018  (DROPLET) | - | 2011 UK census (White British/White Irish/White Gypsy or Irish Traveller/Other White background/White + Black Caribbean/White + Black African/White + Asian/Any other mixed or multiple ethnic background/Indian/ Pakistani/Bangladeshi/ Chinese/African/ Caribbean/Other Black, African or Caribbean background/Arab/Any other ethnic group | - | Sex (Female/ Male) | - | - | - | English Indices of Multiple Deprivation [IMD] 2015 (deciles) | Age (continuous) |
| Anderson 2021  (ActWELL) | - | 2011 UK census (White British/White Irish/ Mixed other/Mixed/ Indian/Pakistani/ Chinese/Asian other/ African Caribbean or Black/Other) | Employment (Retired/Unemployed/ Employed full-time/ Employed part-time/ Student full-time/ Student part-time/ Other) | Sex (Female) | - | Highest qualification obtained (Secondary/ Other professional or technical/ University degree) | - | Scottish Index of Multiple Deprivation [SIMD] 2016 | Age (continuous) |
| *Harmonised variable* | ***Urban vs rural*** | ***White (including white minorities)/ Ethnic minority*** | ***Employed/ Unemployed/ Retired/ Student/ Other (sick or unable to work)*** | ***Female/ Male*** | *No data* | ***University degree/ Some additional training/ A-levels or equivalent/ GCSEs, O-levels or equivalent/ No formal qualifications*** | ***(Marital status) Single/ Married, civil partnership or cohabiting/ Divorced, separated or widowed*** | ***Quintiles of IMD (1=most deprived – 5=least deprived)***  ***Household income:***  ***>£40,000 / <£40,000*** | ***Age (continuous)*** |

# Supplementary Table 4 Summary of participants included in complete case analyses where individual participant data were accessed

| Characteristic | N (%) |
| --- | --- |
| Place of residence (General Practice location) *n=1 study* | |
| Urban | 240 (83.6) |
| Rural | 47 (16.4) |
| Ethnicity *n=7 studies* | |
| White | 4233 (95.6) |
| Ethnic minority | 198 (4.4) |
| Occupation *n=5 studies* | |
| Employed | 907 (51.2) |
| Unemployed | 150 (8.5) |
| Retired | 621 (35.1) |
| Student | 9 (0.5) |
| Other | 83 (4.7) |
| Gender/sex *n=10 studies* | |
| Female | 2479 (59.2) |
| Male | 1712 (40.8) |
| Education *n=7 studies* | |
| University | 834 (34.2) |
| Post-secondary/equivalent | 546 (22.4) |
| A-Levels/equivalent | 479 (19.6) |
| GCSEs/equivalent | 448 (18.4) |
| No formal qualifications | 131 (5.4) |
| Socioeconomic status (IMD or Scottish IMD) *n=9 studies* | |
| 1 | 490 (10.2) |
| 2 | 678 (14.1) |
| 3 | 924 (19.2) |
| 4 | 1198 (24.8) |
| 5 | 1534 (31.8) |
| Socioeconomic status (annual household income) *n=2 studies* | |
| >£40,000 | 651 (71.0) |
| <£40,000 | 266 (29.0) |
| Social Capital *n=2 studies* | |
| Married/cohabiting | 1117 (86.5) |
| Single | 71 (5.5) |
| Separated/widowed/divorced | 103 (8.0) |

# Supplementary Table 5 Percentage difference between groups for intervention attendance

| Characteristic | Percentage difference in attendance (95% confidence interval) | I^2^ | Tau^2^ | P-value |
| --- | --- | --- | --- | --- |
| Ethnicity *n=3 studies* | | | | |
| White vs Ethnic minority (Excluding White minorities) | 3.47 (-8.46, 15.39) | 71.68% | 95.42 | 0.57 |
| Occupation *n=3 studies* | | | | |
| Unemployed (vs Employed) | -1.88 (-9.56, 12.92) | 0.00% | 0.00 | 0.63 |
| Retired (vs Employed) | -2.30 (-9.17, 4.57) | 35.22% | 13.10 | 0.51 |
| Student (vs Employed) | -7.24 (-20.14, 5.65) | 0.00% | 0.00 | 0.27 |
| Other (vs Employed) | 1.45 (-4.30, 7.20) | 0.00% | 0.00 | 0.62 |
| Gender/sex *n=4 studies* | | | | |
| Male (vs female) | -1.85 (-5.47, 1.76) | 0.00% | 0.00 | 0.31 |
| Education *n=3 studies* | | | | |
| Post-secondary (vs University) | -6.05 (-13.68, 1.57) | 0.00% | 0.00 | 0.12 |
| A-Levels/equivalent (vs University) | -1.88 (-7.91, 4.15) | 0.00% | 0.00 | 0.54 |
| GCSEs/equivalent (vs University) | -0.97 (-6.30, 4.36) | 0.00% | 0.00 | 0.72 |
| No formal qualifications (vs University) | -3.66 (-25.08, 17.76) | 83.71% | 199.97 | 0.74 |
| Socioeconomic status (IMD) *n=4 studies* | | | | |
| IMD 2 (vs 1) | -4.67 (-34.92, 25.57) | 98.88% | 792.60 | 0.76 |
| IMD 3 (vs 1) | 0.12 (-0.63, 0.87) | 0.00% | 0.00 | 0.75 |
| IMD 4 (vs 1) | 2.75 (-4.33, 9.82) | 54.79 | 26.10 | 0.45 |
| IMD 5 (vs 1) | 5.82 (-3.05, 14.68) | 55.13% | 38.85 | 0.20 |
| Socioeconomic status (annual household income) *n=1 study* |  |  |  |  |
| >£40,000 (vs <£40,000) | 6.34 (0.812, 12.62) | n/a* | n/a* | 0.04 |
| Social capital (marital status) *n=1 study* |  |  |  |  |
| Single (vs married or cohabiting) | -4.28 (-16.87, 8.31) | n/a* | n/a* | 0.50 |
| Separated, widowed or divorced (vs married or cohabiting) | -5.61 (-15.90, 4.68) | n/a* | n/a* | 0.29 |
| Age *n=5 studies* | | | | |
| Continuous (coefficient refers to 1-year increase in age at baseline) | 0.12 (-0.14, 0.38) | 69.74% | 0.05 | 0.36 |

*Footnote: * Denotes not available as data came from one study, so was not included in any meta-analyses*

# Supplementary Table 6 Difference in weight at 12-months across the cohort of participants in trials of behavioural weight management interventions

| Characteristic | Difference in weight at 12 months in kilograms  (95% confidence interval) | I^2^ | Tau^2^  (95% prediction interval) | p-value of association |
| --- | --- | --- | --- | --- |
| Place of residence (General Practice location) *n=1 study* |  |  |  |  |
| Rural (vs Urban) | 0.22 (-1.43, 1.87) | n/a* | n/a* | 0.79 |
| Ethnicity *n=8 studies* |  |  |  |  |
| Ethnic minority (vs White (including White minorities)) | 0.46 (-0.45, 1.37) | 26.73% | 0.50 (-1.49, 2.41) | 0.32 |
| Occupation *n=6 studies* | | | | |
| Unemployed (vs Employed) | -0.64 (-1.86, 0.59) | 0.00% | 0.00 (-2.64, 1.36) | 0.31 |
| Retired (vs Employed) | -0.65 (-1.50, 0.21) | 0.00% | 0.00 (-2.54, 1.24) | 0.14 |
| Student (vs Employed) | 2.44 (-0.46, 5.34) | 0.00% | 0.00 (-3.92, 8.81) | 0.10 |
| Other (vs Employed) | 1.05 (-0.16, 2.26) | 43.41 | 0.80 (-0.91, 3.01) | 0.09 |
| Gender/sex *n=11 studies* | | | | |
| Male (vs Female) | 0.62 (0.10, 1.15) | 27.13% | 0.20 (-0.56, 1.80) | 0.02 |
| Education *n=8 studies* | | | | |
| Post-secondary (vs University) | 0.35 (-0.35, 1.05) | 3.71% | 0.03 (-0.91, 1.61) | 0.33 |
| A-Levels/equivalent (vs University) | -0.03 (-0.76, 0.7) | 9.32% | 0.08 (-1.06, 1.00) | 0.93 |
| GCSEs/equivalent (vs University) | 0.43 (-0.43, 1.30) | 0.00% | 0.00 (-1.48, 2.34) | 0.33 |
| No formal qualifications (vs University) | 1.45 (-0.10, 2.99) | 21.87% | 0.68 (-1.05, 3.95) | 0.07 |
| Socioeconomic status (IMD or Scottish IMD) *n=10 studies* | | | | |
| IMD Quintile 2 vs 1 (most deprived) | 0.05 (-0.59, 0.70) | 0.00% | 0.00 (-0.71, 0.81) | 0.87 |
| IMD Quintile 3 vs 1 | 0.08 (-0.57, 0.72) | 0.00% | 0.00 (-0.67, 0.83) | 0.81 |
| IMD Quintile 4 vs 1 | -0.39 (-1.05, 0.26) | 0.00% | 0.00 (-1.15, 0.37) | 0.24 |
| IMD Quintile 5 (least deprived) vs 1 | -0.26 (-0.98, 0.47) | 10.72% | 0.15 (-1.12, 0.60) | 0.49 |
| Socioeconomic status (annual household income) *n=2 studies* |  |  |  |  |
| >£40,000 (vs <£40,000) | -0.38 (-1.29, 0.53) | 0.00% | 0.00 (n/a^) | 0.41 |
| Social Capital *n=2 studies* |  |  |  |  |
| Single (vs Married/cohabiting) | 0.61 (-0.76, 1.99) | 0.00% | 0.00 (n/a^) | 0.38 |
| Separated/widowed/divorced (vs Married/cohabiting) | 1.31 (0.18, 2.43) | 0.00% | 0.00 (n/a^) | 0.02 |
| Age *n=11 studies* | | | | |
| Continuous (coefficient refers to one-year increase in age at baseline) | -0.05 (-0.07, -0.04) | 0.00% | 0.00 (-0.06, -0.04) | <0.01 |


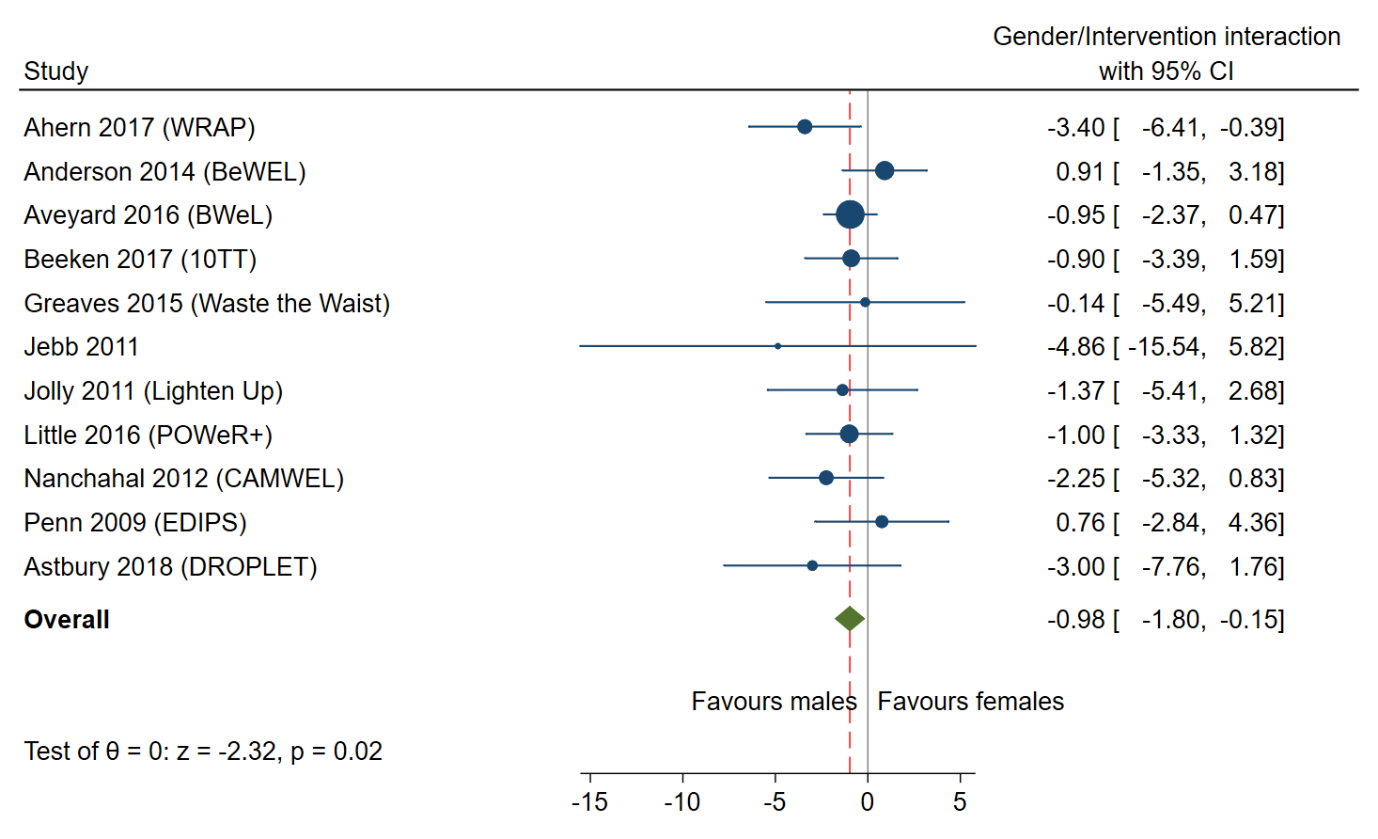


# Supplementary Figure 1 Forest plot of the difference (male minus female) in mean differences between the intervention and control groups for gender/sex


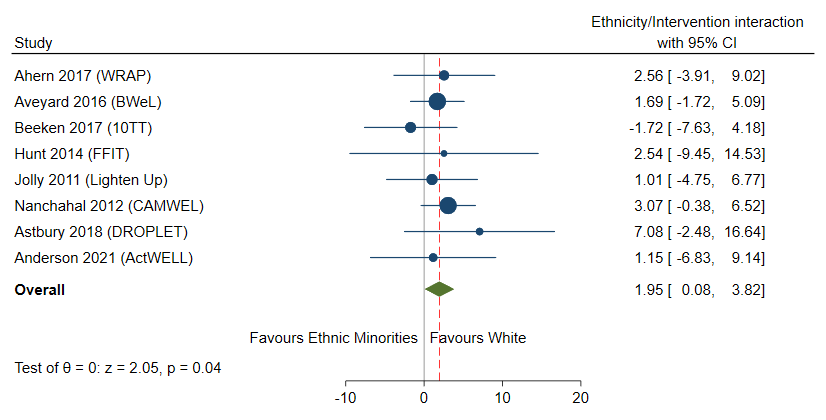


# Supplementary Figure 2 Forest plot of the difference (White minus Ethnic Minority) in mean differences between the intervention and control groups for ethnicity
